# Radium-223-Dichloride in Castration Resistant Metastatic Prostate Cancer—Preliminary Results of the Response Evaluation Using F-18-Fluoride PET/CT

**DOI:** 10.3390/diagnostics5040413

**Published:** 2015-10-13

**Authors:** Kalevi Kairemo, Timo Joensuu

**Affiliations:** 1 Departments of Molecular Radiotherapy & Nuclear Medicine, Docrates Cancer Center, Saukonpaadenranta 2, Helsinki FI-00180, Finland; 2 Departments of Radiotherapy and Medical Oncology, Docrates Cancer Center, Saukonpaadenranta 2, Helsinki FI-00180, Finland; E-Mail: timo.joensuu@docrates.com

**Keywords:** prostate cancer, sodium fluoride, skeletal metastases, radium-223-dichloride

## Abstract

The purpose of this study was to evaluate the outcome after Radium-223-dichloride (^223^RaCl_2_) treatment of patients with skeletal metastases of castration resistant prostate cancer using whole-body ^18^F-Fluoride PET/CT. Sodium ^18^F-fluoride [^18^F]-NaF PET/CT was performed prior the treatment of ^223^RaCl_2_, after the first cycle and after the sixth cycle. The skeletal metastases were analyzed quantitatively using modified PET response evaluation PERCIST criteria. The patients were also analyzed for S-PSA. All ten patients responded in [^18^F]-NaF scans after 6 cycles, but interim analysis after the 1st cycle did not give additional information about the outcome. The S-PSA decrease correlated with [^18^F]-NaF response, only 1 patient demonstrated progressive disease, *i.e*., >25% increase in S-PSA values during ^223^RaCl_2_. Our results (although preliminary) suggest that ^18^F-Fluoride PET/CT is useful in the follow-up of castration resistant prostate cancer with skeletal metastases.

## 1. Introduction

Prostate cancer is the most common cancer in Europe and 10%–20% of patients present with advanced or metastatic disease with associated symptomatic bone metastases [1]. Bone is the most frequent metastatic site in prostate cancer, with approximately 90% of patients with metastatic castration resistant prostate cancer (mCRPC) having radiological evidence of bone metastases [2]. Bone metastases profoundly affect an individual’s quality of life, increase the risk of bone marrow failure [3] and skeletal-related events (SREs) such as pathological fractures and spinal cord compression [4], and significantly reduce life expectancy [5,6]. The main cause of disability and death among those with mCRPC is bone metastases.

According to EANM guidelines, patients considered for therapy with beta-emitting radiopharmaceuticals such as ^89^Sr-Cl, ^153^Sm-EDTMP or ^186^Re-HEDP therapy had to fail in conventional analgesics and anti-tumor therapy, chemotherapy or hormone therapy. Pain had to be severe enough to limit normal activities and/or require regular analgesics, and patients had to undergo recent (within 8 weeks or less) bone scintigraphy documenting increased osteoblastic activity at painful sites. These radionuclide treatments are palliative, where 60%–80% of patients benefit from the treatment and they do not cure metastatic cancer [7,8]. All of these agents have demonstrated improved overall survival in retrospective controlled studies [9,10,11], but there are not yet randomized controlled studies which support these findings, only studies which do not improve survival [12]. In 2010’s, the bone-seeking radiopharmaceutical, radium-223 dichloride (^223^RaCl_2_), was introduced into clinical practice. ^223^RaCl_2_ is indicated for the treatment of patients with mCRPC and symptomatic bone metastases, offering overall survival benefits comparable to the other new treatment options available [13,14,15]. The availability of ^223^RaCl_2_ has generated considerable interest amongst the nuclear medicine community, and in many centers around the world nuclear medicine physicians have joined the multidisciplinary treatment team involved in the management of patients with mCRPC.

This ^223^RaCl_2_ is an alpha-emitting bone targeting agent, which has favorable pharmacokinetic and pharmacodynamic characteristics: at 10 min 12%, at 1 h 6% and at 24 h <1% of the injected activity is in the blood and the skeletal uptake is 44%–77% at 4 h. Fecal excretion is the major elimination route, but additionally 5% is excreted in the urinary tract [15,16]. In regards to diagnostic imaging of these patients, the Society for Nuclear Medicine published guidelines for [^18^F]-NaF PET/CT bone scans [17]. Bone uptake of ^18^F-NaF reflects bone remodeling, and it is part of the mineralization of bone matrix. [^18^F^−^] ion is exchanged for [OH^−^] so that hydroxy apatite bone matrix is transformed into fluoro apatite, indicating that high uptake of [^18^F]-NaF reflects bone reactions to bone injury, not to prostate cancer. Positive findings with [^18^F]-NaF PET/CT are due to both benign and malignant bone disorders. In the literature, ten articles described the use of [^18^F]-NaF PET/CT scans in the diagnosis of metastatic prostate cancer [18,19,20,21,22,23,24,25,26,27]. In our own meta-analysis about [^18^F]-NaF PET/CT in 3918 patients [28], 1289 (33%) had positive scans. Use of [^18^F]-NaF and ^18^F-Choline PET/CT had similar diagnostic accuracy at staging for patients with prostate cancer but ^18^F-Choline has higher specificity at restaging for recurrence [28].

In this article, the outcome of ^223^RaCl_2_ treatment was analyzed using [^18^F]-NaF-PET.

## 2. Methods and Patients

**Patients**: Prostate cancers were diagnosed between 2001 and 2013. In this retrospective one institute analysis we included all those patients who had received at least 6 cycles of ^223^RaCl_2_ therapy. Men’s ages ranged from 49 to 83 years; Gleason scores were 6–10; initial PSA values varied from 5.5 to 15,500 μg/L, and all patients had T3-T4 disease. All the patients had skeletal metastases, and two of them additionally had visceral metastases. The radiological TNM staging before ^223^RaCl_2_ was performed using [^18^F]-NaF-PET and [^18^F]-fluorocholine-PET in all patients, most (9 out of 10) of the patients had also pelvic MRI and diagnostic whole body CT. The ^223^RaCl_2_ injections were given every 4 weeks and follow up studies were done with [^18^F]-NaF. Two of them had previous surgery, 8 had previous radiotherapy, all 10 had androgen deprivation therapy (ADT) as well having received chemotherapy, 2 had received Sm-153-EDTMP therapy, and 9 additionally received denosumab prior ^223^RaCl_2_. The patient characteristics are summarized in Table 1.

This work is a retrospective treatment analysis of our ten first Ra-223 patients followed with [^18^F]-NaF PET/CT imagings. This retrospective analysis was performed, according to the principles of the *Declaration of Helsinki* and our patient database was approved by the Finnish authority for the protection of privacy and personal data. All patients gave a written informed consent for the use their data.

**Imaging PET/CT protocol**: Imaging was done on Siemens Biograph PET Scanner, combined with low-dose CT. The injected activity dose of [^18^F]-NaF ranged 212–290 MBq. Whole body imaging was performed by starting at 60 ± 3.0 min (range 58–76 min) from the calvarium to the tips of the toes using 2.5 min per bed position. All analyzed patients were treated with 6 cycles of radium-223-dichloride (^223^RaCl_2_) and [^18^F]-NaF PET/CT imaging was applied before Ra-223 therapy in all patients, in 6 patients after the 1st cycle and in all patients after the last 6th cycle (Table 2). In one patient (no. 6) there was a production failure of ^223^RaCl_2_ and the interval between the 1st and 2nd cycle was 10 weeks instead of normal 4 weeks. He was imaged after the 2nd cycle, and received a total of 7 cycles. The [^18^F]-NaF after the first (second) cycle was performed 3 weeks after ^223^RaCl_2_ infusion. The last control was performed within 4 weeks after the last 6th (7th) cycle in all patients. The tracer [^18^F]-NaF was provided from MAP Medical Technologies Oy (Helsinki, Finland).

**Alpha-therapy:** ^223^RaCl_2_ (Xofigo^®^) was acquired from Bayer Healthcare AG (Berlin, Germany), each single-use vial contained 6 mL of solution at a concentration of 1000 kBq/mL (27 μCi/mL) and a total radioactivity of 6 MBq/vial (162 μCi/vial) at the reference date [29]. The used activity dose of ^223^RaCl_2_ is 50 kBq/kg body weight (1.35 μCi/kg) given at 4-weekly intervals for six cycles, the cumulative activities are listed in Table 2. The volume administered was calculated using the patient’s body weight (kg), the dosage level (50 kBq/kg body weight), the radioactivity concentration of the product at the reference date, and the decay correction factor provided with each vial.

Prior to the first dose, patients were analyzed for a full blood count (FBC) to assess bone marrow function. Treatment was given if: haemoglobin level >10 g/dL, absolute neutrophil count (ANC) ≥1.5 × 10^9^/L, and platelet count ≥100 × 10^9^/L. Due to the potential myelotoxic effects of treatment, a FBC was undertaken prior to all subsequent treatment cycles. The decision to administer the next cycle depended on recovery of the ANC and platelet counts to levels of ≥1.5 × 10^9^/L and ≥100 × 10^9^/L, respectively. The cumulative doses in 6 cycles varied from 16.45 to 31.57 MBq.

**Table 1:** The patient characteristics.

Patient Age	Diagnosis Gleason Score, TNM (Year)	Distribution (before Ra-223)	Dx/modality *	Cycles * Cumulative Activity	Initial S-PSA	Other Treatments before Xofigo
1 m/63	GS 9, T4N1M1 (-13)	Prostate Bone lnn	NaF, FCH, MRI, CT	6 30.86 MBq	410	Degarelix, Bicalutamide, Docetaxel, Abiraterone, Enzalutamide, Denosumab
2 m/77	GS 7, pT3N0M0 (-01)	Bone	NaF, FCH, FACBC, MRI, CT	6 24.80 MBq	5.5	RRP, EBRT (PO), Bicalutamide, Docetaxel, Abiraterone, Enzalutamide, Denosumab, Goserelin
3 m/68	GS 8, T4N1M1 (-13)	Bone	NaF, FCH, MRI, CT	6 27.08 MBq	15,500	Degarelix,Bicalutamide, Denosumab, EBRT (B), Docetaxel, Abiraterone, EBRT (P), Enzalutamide, Denosumab, Goserelin
4 m/49	GS 9, T4N1M1 (-13)	Bone	NaF, FCH, MRI, CT	6 31.57 MBq	700	Cyproterone, Degarelix, Bicalutamide, Zoledronate, Docetaxel, Sm-153-EDTMP, Mitoxantrone, EBRT (P), Docetaxel, Abiraterone
5 m/67	GS 9, T3N1M1 (-13)	Bone	NaF, FCH, MRI, CT	6 25.87 MBq	430	Degarelix, Bicalutamide, Denosumab, Docetaxel, Goserelin, Enzalutamide
6 m/69	GS 8, T4N1M1 (-11)	Bone	NaF, FCH, MRI, CT	7 24.13 MBq	16.2	Bicalutamide, Goserelin, EBRT (P), Abiraterone, Denosumab, Docetaxel, Enzalutamide
7 m/56	GS 10, T4NxM1 (-13)	Bone	NaF, FCH, MRI, CT	6 22.78 MBq	790	Bicalutamide, Lupron, Denosumab, EBRT (P), Sm-153-EDTMP, Mitoxantrone, Docetaxel, Abiraterone
8 m/58	GS 9, T3N0M1 (-12)	Bone	NaF, FCH, MRI, CT	6 23.71MBq	6.5	Denosumab, Degarelix, Bicalutamide, EBRT (P), Goserelin, Docetaxel
9 m/60	GS 9, T4M1N1 (-12)	Bone	BS, NaF, FCH, MRI, CT	6 16.45 MBq	18	Bicalutamide, Buserelin, TURP, Docetaxel, EBRT (B), Abiraterone, Denosumab, Cabazitaxel, Enzalutamide
10 m/83	GS 6, T3NxM0 (-01)	Bone Liver lnn	NaF, FCH	6 24.06 MBq	18	Leuprolin, EBRT (P), Docetaxel, Abiraterone, Mitoxantrone, Zoledronate, Denosumab

* BS = bone scintigraphy, CT = computer tomography, FACBC = F-18-ACBC PET/CT, FCH = F-18-choline PET/CT, MRI = magnetic resonance imaging, NaF = F-18-fluoride PET/CT; Abbreviations: P = radical radiotherapy of prostate, B = radiotherapy of bone metastases, PO = postoperative radiotherapy, lnn = lymph nodes, GS = Gleason score.

**Image analysis:** Lesions were considered abnormal when focal tracer accumulation was greater than background activity, usually if the SUV values were higher than 10.

Interpretation of bone lesions (benign or malignant) depended on anatomical localization; and the five regions: skull, vertebral column, thoracic girdle, pelvic girdle and extremities were estimated quantitatively using modified PERCIST criteria for NaF. In the classic PERCIST analysis, the skeleton is considered as organ, and a maximum of two lesions per organ are taken into account out of the total sum of five lesions. Therefore, here two highest SUVmax values of skeletal uptakes from two regions were summed. The results were compared with those of base line, the change of 6% were considered significant as in conventional PERCIST consisting of 5 lesions, where 15% change is considered significant which is analogous to PERCIST criteria for early response [30].

**Table 2:** The summary of the NaF imaging results.

Patient Age	Bone Distribution # (before Ra-223)	NaF-% Change after 1st/ 6th Cycle (2nd/7th)	Number of Cycles Cumulative Activity	S-PSA Response	Treatments during Xofigo
1 m/63	Bone 3/3	+0.8/−13.8	6 30.86 MBq	PD	Degarelix, Enzalutamide, Denosumab
2 m/77	Bone 2/3	+6.2/−6.9	6 24.80 MBq	PR	Enzalutamide, Denosumab, Goserelin
3 m/68	Bone 3/3	−10.8/−9.2	6 27.08 MBq	CR	Enzalutamide, Denosumab, Goserelin
4 m/49	Bone 3/3	nd/−13.0	6 31.57 MBq	PD/SD	Lupron,Denosumab, Abiraterone
5 m/67	Bone 1/3	−6.5/−7.8	6 25.87 MBq	PR	Denosumab, Goserelin, Enzalutamide
6 m/69	Bone 3/3	−10.4/−11.7	7 24.13 MBq	PR	Goserelin, Denosumab, Enzalutamide
7 m/56	Bone 3/3	nd/−11.4	6 22.78 MBq	PR	Lupron, Denosumab, Abiraterone
8 m/58	Bone 3/3	nd/−43.0	6 23.71MBq	SD/PD	Denosumab, Degarelix, Abiraterone
9 m/60	Bone 2/3	nd/−68.4	6 16.45 MBq	SD	Buserelin, Denosumab, Enzalutamide
10 m/83	Bone 1/3	+6.3/−63.5	6 24.06 MBq	PR	Denosumab, Degarelix, Enzalutamide

**#** 1/3 10%–25% of the skeleton affected; 2/3 25%–50% of the skeleton affected; 3/3 >50% of the skeleton affected. Abbreviations: CR =complete response, PD = progressive diseased, PR = partial response, SD = stable disease; nd = not done.

**Statistical analysis:** The acquired results were expressed as the mean ± SEM for each index. Comparison of data among various groups was performed with Student’s unpaired *t*-test. A *p* < 0.05 was considered statistically significant. Correlation between groups was calculated using linear regression analysis.

## 3. Results

**Figure 1:**
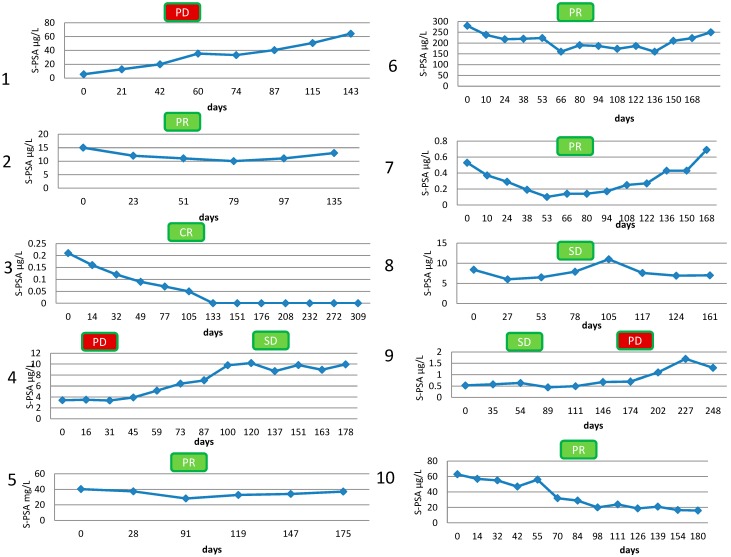
PSA-curves before, during 6 cycles, and 1 month after Ra-223 treatments in all patients; response, if >25% decrease in S-PSA values. SD = stable disease, PR = partial response (>25% decrease), CR = complete response (>75% decrease), PD = progressive disease (>25% increase). The number refers to patient number (Table 1 and Table 2), y-axis S-PSA concentration in μg/L, x-axis time in days, starting from the day of 1st Ra-223 treatment. Numbers 1–10 refer to patient number in Table 1, Table 2 .

The summary of imaging results is presented in Table 2. All patients responded to their treatment according to the [^18^F]-NaF imaging data. Their PSA-curves are shown in Figure 1. Partial response, *i.e*., 25%–75% decrease in S-PSA values was seen 5 patients. One patient demonstrated complete response, *i.e*., S-PSA values were = 0.0 or decreased >75%. Stable disease <25% decrease or increase was seen in 3 patients and 1 patient had progressive disease.

**Figure 2:**
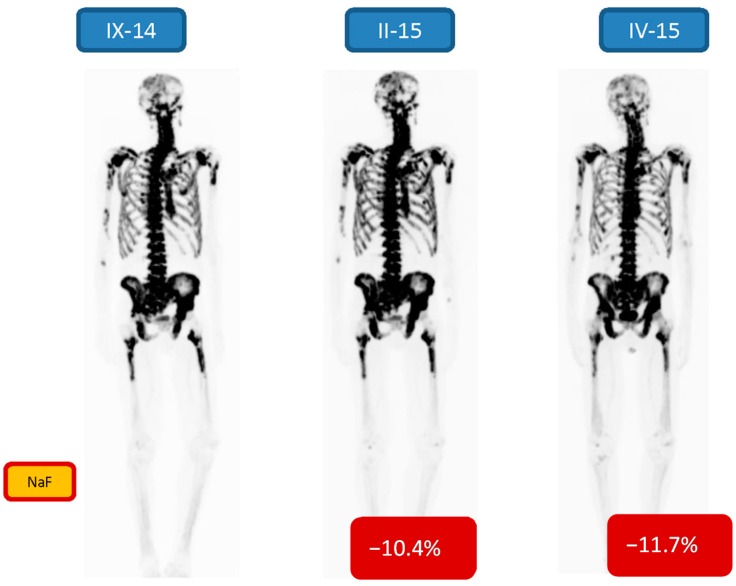
69-year old male, Gleason score 8 (diagnosed in 2012), treated earlier with Casodex, Zoladex, S-PSA 671 ng/mL at initial visit in (VII/14), was treated with 6 cycles of ^223^RaCl_2_. A NaF-PET-CT study before (IX-14), one interim study (after two cycles, II-15) and one study (IV-15) after the 6 six cycles are shown. There is a quantitative response (−10.4% and −11.7%) in both the interim and post therapy NaF-PET-maximum intensity projection images.

A few patient examples with imaging data are shown. Figure 2 demonstrates the [^18^F]-NaF imagings before, one interim analysis (after two cycles) and one evaluation after the 6 six cycles in a 69-year old male (patient no. 6). His Gleason score was 8 (diagnosed in 2012), treated earlier with Casodex, Zoladex, PSA 671 μg/L at initial visit (in VII/14), and he was treated with 6 cycles of ^223^RaCl_2_. Three evaluations are shown: A [^18^F]-NaF-PET-CT before, one interim (after two cycles) and one after the 6 six cycles of Ra-223. There were quantitative changes (−10.4% and −11.7%) in both the interim and post therapy NaF-PET. The PSA-curve demonstrated partial response (6, Figure 1), but the ALP curve clearly demonstrated the delay in response due to ^223^RaCl_2_ production failure (interval between 1. and 2. cycle was 10 weeks).

**Figure 3:**
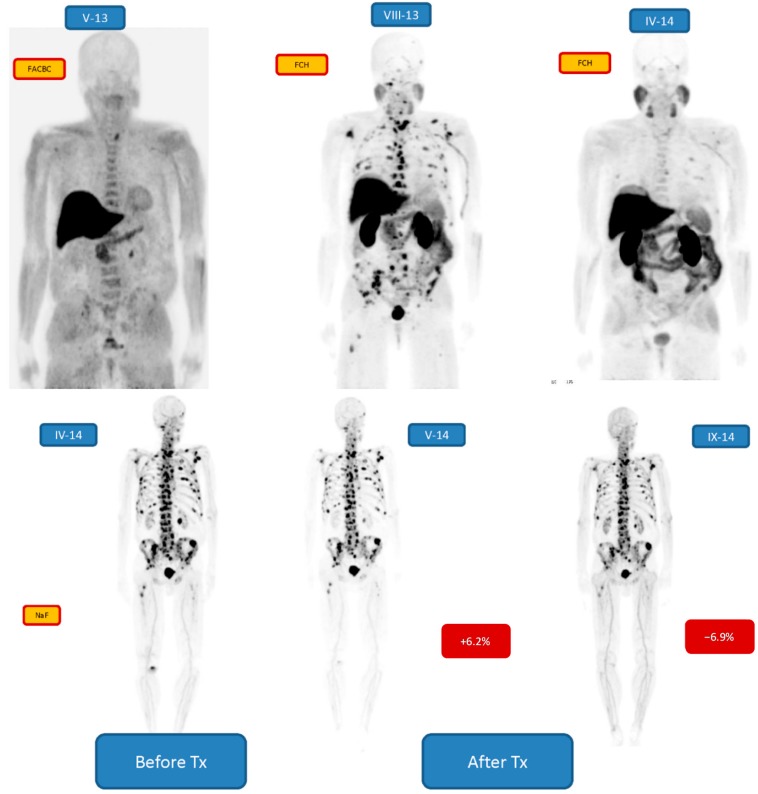
Demonstrates a patient with Gleason score 7 treated originally with radical prostatectomy in 2001, treated subsequently with external beam radiation therapy, then with bicalutamide, docetaxel, abiraterone, enzalutamide, denosumab, doserelin. He demonstrated a biochemical relapse with a few findings on FACBC-PET (V-13), but with more findings on FCH-PET 3 months later (VIII-13). The response to the treatment was seen on FCH –PET in 8 months (IV-14), where the changes in the bone marrow had dramatically disappeared. However, NaF-PET on the following day (IV-14) demonstrated a widespread active cortical bone disease. This did not respond to the treatment after 1st cycle (+6.2% change, V-14), but after 6 cycles a response was seen (−6.2% change, IX-14).

Figure 3 demonstrates another patient (no. 2) with Gleason score 7 treated with radical prostatectomy, (diagnosed in 2001), and with postoperative external beam radiation therapy, then with bicalutamide, docetaxel, abiraterone, enzalutamide, denosumab and goserelin. He demonstrated a biochemical relapse with a few findings on [^18^F]-FACBC-PET-CT [31], but with more findings on [^18^F]-FCH-PET-CT 3 months later. The response to the treatment was seen on [^18^F]-FCH-PET in 8 months, where the changes in the bone marrow had dramatically disappeared. However, [^18^F]-NaF-PET on the following day demonstrated a widespread active cortical bone disease. This did not respond to the treatment after 1st cycle (+6.2% change), but after 6 cycles a minor response was seen (−6.2% change).

**Figure 4:**
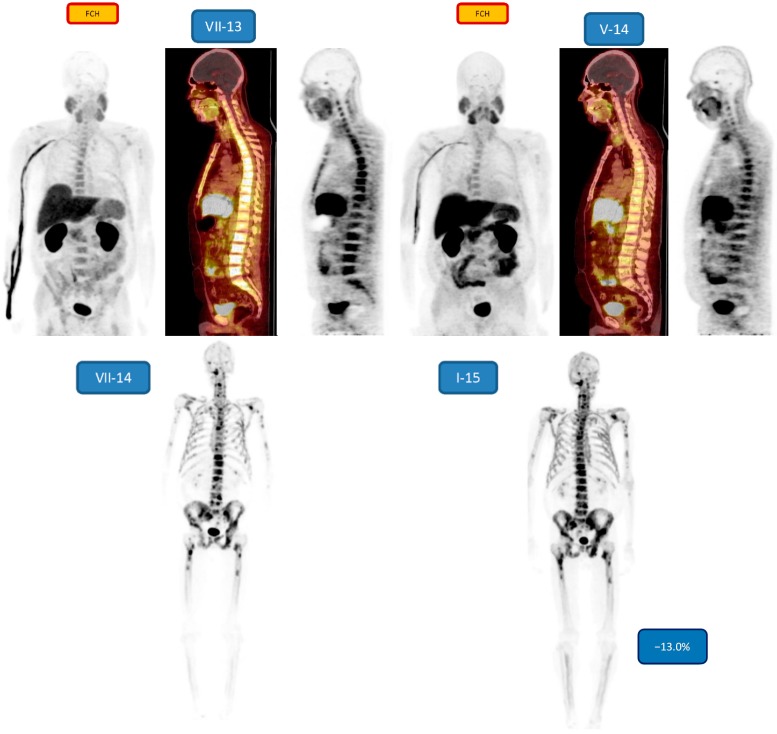
Demonstrates a young patient (49 years) with Gleason score 9, treated earlier with cyproterone, Degarelix, bicalutamide, zoledronate, docetaxel, Sm-153-EDTMP, mitoxantrone, EBRT (P), docetaxel, abiraterone. A response to the treatments is seen on FCH-PET in 10 months, especially in the bone marrow disease, MIP-image, sagittal fusion FCH-PET on CT and sagittal FCH-PET figures are shown in July 2013 (VII-13) and corresponding data in May 2014 (V-14). NaF-PET 6 weeks (VII-14) later in July 2014 demonstrated a widespread active cortical bone disease. This responded to the Ra-223 treatment, because after 6 cycles a response was seen in January 2015 (−13.0% change, I-15).

Figure 4 demonstrates a young patient (49 years, patient no. 4) with Gleason score 9, treated earlier with cyproterone, degarelix, bicalutamide, zoledronate, docetaxel, Sm-153-EDTMP, mitoxantrone, EBRT (P), docetaxel, abiraterone.

A response to the treatments is seen on [^18^F]-FCH–PET in 10 months, especially in the bone marrow disease.

[^18^F]-NaF-PET 6 weeks later demonstrated a widespread active cortical bone disease. This responded to the Ra-223 treatment, because after 6 cycles a response was seen (−13.0% change).

**Figure 5:**
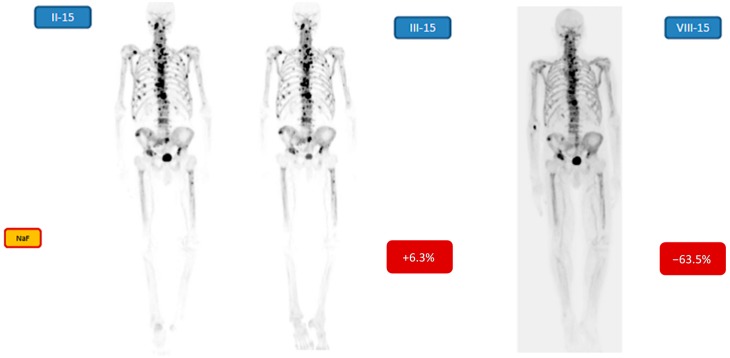
Demonstrates an old patient (83 years) with Gleason score 6. The NaF-PET before the Ra-223 treatment 6 cycles is shown (II-15), but no essential response to the treatment after 1st cycle (+6.3% change, III-15) was seen, whereas after 6 cycles a dramatic response was seen (−63.5% change, VIII-15). Many of the lesions have disappeared, especially in the thoracic girdle.

Figure 5 demonstrates an old patient (83 years) with Gleason score 6 (patient no. 10). The [^18^F]-NaF-PET before the Ra-223 treatment is shown, but no essential change in the total [^18^F]-NaF-PET activity due to the Ra-223 treatment was seen after 1st cycle (+6.3% change), whereas after 6 cycles a dramatic response was seen (−63.5% change).

## 4. Discussion

Our patients with active mCRPC all responded to their treatments, because quantitatively there was a significant decrease in [^18^F]-NaF-PET findings after 6 cycles of ^223^RaCl_2_ as compared to the baseline measurements. The quantitative decrement varied from −6.9% to −68.4%. The change can be visually seen in Figure 5 (quantitatively −63.5%), but no so easily in Figure 2, Figure 3 and Figure 4 (quantitatively −6.9% to 13.0%). Therefore, quantification is important in widespread, *i.e.* more than 10% of skeleton, metastatic bone disease. According to the S-PSA measurements all patients except one (patient no. 1) showed some response in an active aggressive (T3-T4) widespread skeletal disease.

There are now multiple new therapies providing options for the treatment of patients having metastatic castration-resistant prostate cancer (mCRPC). For these patients, therapies were just very recently exceptionally ineffective and extremely limited to the use of docetaxel. These new therapies include androgen synthesis inhibitor (abiraterone) [32], androgen receptor signaling inhibitor (enzalutamide) [33], radionuclide therapy (radium-223) [34,35,36,37] and cabazitaxel chemotherapy [38].

Radium-223 was the first bone-seeking radionuclide that is reported to increase overall survival, and quality of life by providing pain relief in their late stage disease from symptomatic skeletal events (SSEs) such as bone pain, pathological fractures, or spinal cord compression seen in up to 90% of mCRPC patients. This new drug, radium-223-dichloride (^223^RaCl_2_) is a new bone-seeking calcium analogue alpha-emitter, first of its kind in clinical use (Marketing authorization EU/1/13/873/001). It targets increased bone turnover developed by metastatic bone disease. In its double-blinded randomized registration trial, the ^223^RaCl_2_ receiving patients demonstrated median overall survival of 14 months, *vs.* 11.2 months for those on placebo [34,35].

Our patients with active mCRPC all responded to the given therapies. The common denominator was that they all received multimodality treatments with response. All of them received radium-223-dichloride (^223^RaCl_2_) plus denosumab, but otherwise this group of 10 patients was very heterogeneous. This is usually the case in retrospective analysis describing the clinical practice, when it comes to their demographics and previous and concomitant therapies (Table 1 and Table 2).

In addition to the overall survival and QOL benefits, all the secondary endpoints were also met in the Phase 3 ALSYMPCA (ALpharadin in SYMptomatic Prostate CAncer) registration trial [30,31,32,33], which included the delay in time to first skeletal-related events: first events occurred in 13.6 months in the ^223^RaCl_2_ group as compared to 8.4 months in the placebo group—an improvement of 64%. 33% of ^223^RaCl_2_ patients had total alkaline phosphatase normalization as compared to just 1% in the placebo group. Additionally, the ^223^RaCl_2_ patients had an improvement of 49% in time to PSA progression.

Very little, however, is known about the response evaluation of Ra-223 treatment. Although it has been shown that ^223^RaCl_2_ is a bone targeting treatment, its response using bone targeting imaging has not yet been assessed in the literature. There is evidence that NaF-PET can be used as a prognostic factor of the outcome of Ra-223 treatment [39]. They demonstrated that skeletal metastatic burden on baseline will reflect on the outcome of ^223^RaCl_2_ treatment and appearance of skeletal related events. In the PERCIST criteria [30] early minor response is recorded, if there is a 15% decrease in a total sum of 5 lesions; however, a maximum of two lesions can be taken from the same organ. In our modified criteria we therefore selected just the two lesions to represent the whole skeleton. If we use the 30% criteria for partial response as in PERCIST [30], the limit would be 12%, meaning that only 5 of our 10 patients demonstrated response. In the MD Anderson Cancer Center study [40], also the total skeletal volume was analyzed based on SUV-threshold of 10. However, this cannot be applied in the clinical routine, and it does not exclude heterogeneity in the metastatic distribution. It is a challenge to identify the best possible way of interpreting total malignant activity in the skeleton. In this work we wanted to present a simple method for routinely analyzing the overall skeletal situation, and it seems, at least preliminarily, that we have been successful. The threshold has to be settled in a larger patient population.

All our patients were diagnosed with multiple PET tracers which all can be used for assessment of skeletal metastatic distribution. It is obvious, e.g., patient no. 2 who was scanned with [^18^F]-fluorocholine (FCH), [^18^F]-fluciclovine (FACBC) and [^18^F]-NaF, that all these tracers give different information about the *in vivo* biochemistry in prostate cancer. [^18^F]-FCH is an indicator for lipid metabolism and cell membrane synthesis in prostate cancer, [^18^F]-FACBC as an amino acid analog for protein synthesis and [^18^F]-NaF marker for an increased phosphate turnover in reactive osteoblastic cells. In our patients, bone marrow uptake is typical to [^18^F]-FCH, cortical bone uptake for NaF. The distribution for FCH and NaF can be totally different, as shown in Figure 3 on studies on consecutive days.

Flare phenomenon might have been seen in 3 out of 6 patients (Figure 3 and Figure 5), which may indicate that the treatment response analysis after 1st cycle is not useful. The interim [^18^F]-NaF uptake may also be due to disease progression, but the S-PSA values do not support this finding. At least, in this limited material there was no trend in the interim [^18^F]-NaF values after first or second cycle. The [^18^F]-NaF uptake after 6 cycles, however, was significantly lower in all 10 patients as compared to the baseline.

Therefore, we postulate that skeletal response to therapies can be assessed with [^18^F]-NaF. There was also a change in PSA (Figure 1). Response to the therapies can be due to combined effect of abiraterone, enzalutamide or denosumab and Ra-223 alone. Ra-223 and denosumab were the only common regimens in all these therapies. On the other hand Ra-223 can be combined with other therapies with success even though the mechanisms and combinations have to be studied carefully in order to understand the causality.

## 5. Conclusion

Our results suggest that ^18^F-Fluoride PET/CT is the most useful PET method in the follow-up of castration resistant prostate cancer with skeletal metastases. The presented quantification technique suits for response evaluation of ^223^RaCl_2_ in clinical routine.
